# Neuroendocrine Regulation and Metabolism of Glucose and Lipids in Primary Chronic Insomnia: A Prospective Case-Control Study

**DOI:** 10.1371/journal.pone.0061780

**Published:** 2013-04-12

**Authors:** Eleonora Seelig, Ulrich Keller, Markus Klarhöfer, Klaus Scheffler, Serge Brand, Edith Holsboer-Trachsler, Martin Hatzinger, Stefan Bilz

**Affiliations:** 1 Endocrinology, Diabetology and Metabolism, University Hospital Basel, Basel, Switzerland; 2 Institute of Radiology, University Hospital Basel, Basel, Switzerland; 3 MRC Department, Max Planck Institute for Biological Cybernetics, Tübingen, Germany; 4 Center for Affective, Stress and Sleep Disorders, Psychiatric Hospital of the University of Basel, Basel, Switzerland; 5 Endocrinology and Diabetology, Kantonsspital St. Gallen, St. Gallen, Switzerland; Glasgow University, United Kingdom

## Abstract

**Objectives:**

To investigate the relation between primary chronic insomnia and insulin sensitivity, visceral adiposity, non alcoholic fatty liver disease and neuroendocrine hormones.

**Materials and Methods:**

In a case-controlled, prospective clinical trial 13 women with primary chronic insomnia according to DSM-IV criteria were compared to 12 healthy controls matched for age, sex, BMI, body composition and menopausal status. All participants had a sleep assessment including polysomnographic studies and neuropsychiatric evaluation. Insulin sensitivity was evaluated using the euglycaemic hyperinsulinemic clamp. Hepatic fat content, visceral adipose tissue and intramyocellular lipid accumulation were assessed using magnetic resonance imaging and spectroscopy. The hormonal stress axis was evaluated by measurements of midnight and early morning salivary cortisol, urinary catecholamines and plasma metanephrines. Body composition was determined using body impedance analysis and indirect calorimetry.

**Results:**

Although the diagnosis of primary chronic insomnia was made by established clinical criteria, standard polysomongraphic studies failed to identify altered sleep continuity and architecture when compared to matched controls. However, women with primary chronic insomnia showed significantly higher midnight salivary cortisol concentrations (1.46 vs. 0.76 nmol/l, *p = 0.02*), indicating dysregulation of the hypothalamo-pituitary-adrenal (HPA) axis. Plasma glucose and lipid concentrations, insulin sensitivity, hepatic and intramyocellular fat content, visceral adipose tissue mass and body composition did not differ between the two groups.

**Conclusion:**

Healthy women with clinically diagnosed primary chronic insomnia demonstrate a dysregulation of circadian cortisol secretion despite normal sleep continuity and architecture. Increased midnight cortisol levels, however, were not associated with impaired metabolism of glucose and lipids.

## Introduction

Diabetes mellitus type 2 has an increasing prevalence in our society [1], [2]. Changes in dietary habits and diminished physical activity are important contributors to this development. Another behavioral change that has occurred during the same time period is the increased prevalence of sleep curtailment [3]. Both animal and clinical studies have established the importance of an intact circadian rhythm for the regulation of metabolic processes and sleep deprivation has been linked to impaired glucose and lipid metabolism [3], [4], [5], [6], [7], [8]. The identification of so-called clock genes has further highlighted the importance of circadian rhythms for the regulation of metabolic processes. These genes promote the production of downstream transcription factors, which in turn regulate genes involved in metabolic pathways [4]. Interestingly, mice homozygous for a loss of function mutation in the clock genes develop features of the metabolic syndrome [5]. Several clinical studies in humans also suggested a relation between sleep deprivation and insulin resistance. Spiegel et al., for example, showed that short time sleep restriction promoted an impairment of glucose tolerance in healthy young men [6]. Sleep apnea has been identified as a common trait of the metabolic syndrome and its treatment led to improved insulin sensitivity in obese patients [7], [9]. Moreover, a recently published meta-analysis suggested that quantity and quality of sleep predicts the risk of developing diabetes [8]. The impact of acute sleep loss on metabolism has been thoroughly investigated, but the relation between chronic sleep loss and metabolism still needs further analysis [10].

The aim of the present investigation was therefore to assess whether (i) patients diagnosed with primary chronic insomnia show alterations of glucose and lipid metabolism when compared to age, sex and body mass index (BMI)-matched healthy controls, (ii) the potential metabolic alterations could promote the development of features of type 2 diabetes and its associated metabolic disorders, such as dyslipidaemia, visceral adiposity and non-alcoholic fatty liver disease. Moreover, we were interested to see (iii) whether the-pituitary-adrenal axis and the sympathoadrenal system, which play a role in the regulation of glucose metabolism, may be dysregulated in patients with primary insomnia. To this purpose we defined the metabolic alterations in patients with primary insomnia and compared them to age, BMI and menopausal status-matched healthy controls in a case-controlled, prospective trial.

## Materials and Methods

The protocol was approved by the Human Ethics Committee of Basel. All participants gave written informed consent prior to the study (Clinical Trial Registration Number: NCT00442624).

**Figure 1:**
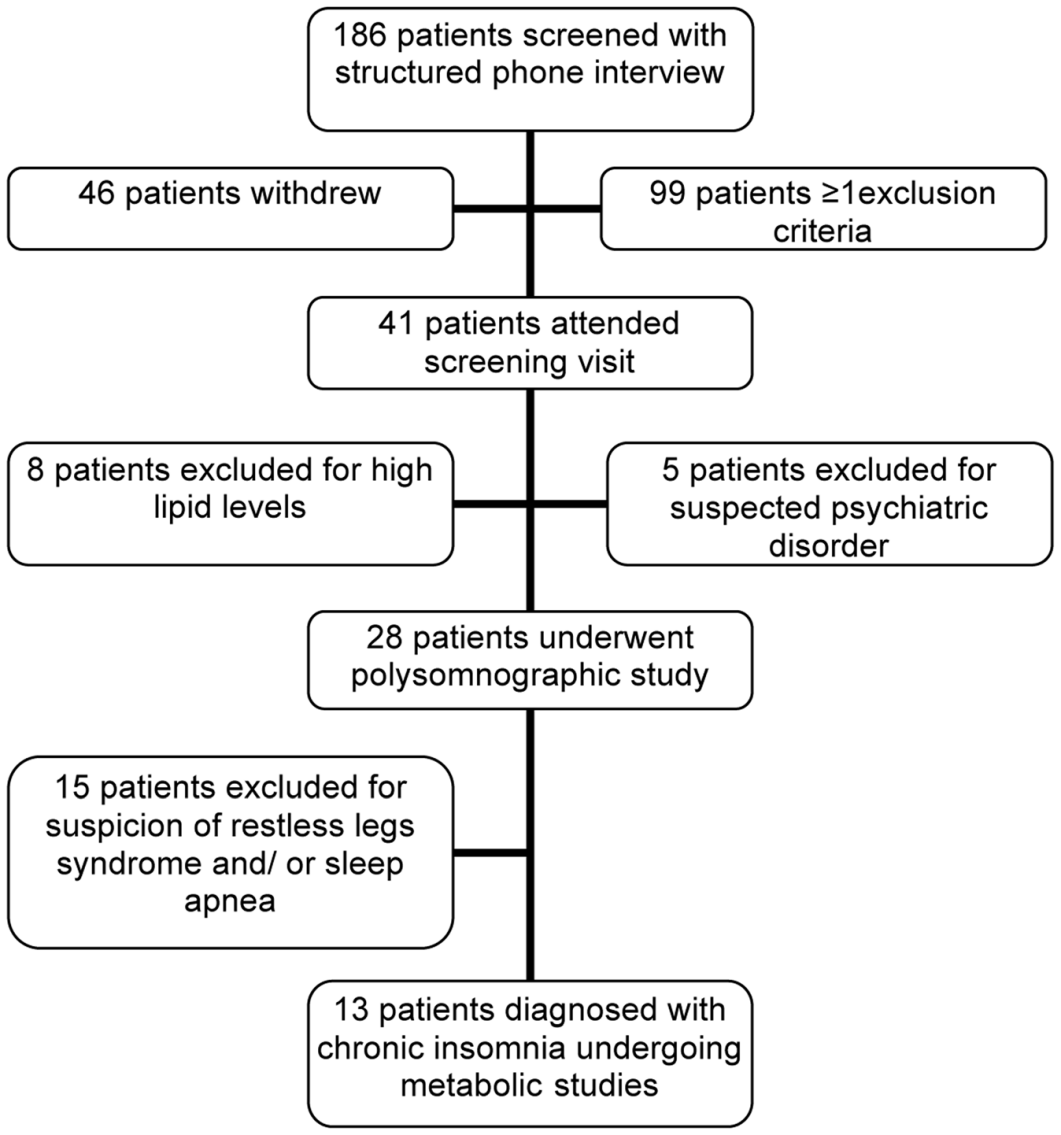
Recruitment of participants.

Patients with primary chronic insomnia were recruited from the Depression and Sleep Research Unit, Psychiatric University Clinics, Basel, and by advertisements in local newspapers. Participants responding to advertisements were screened by a structured telephone interview. Those deemed eligible were invited to join a screening visit in the outpatient department of the Division of Endocrinology, University Hospital Basel (see Fig 1). Written informed consent was obtained from all participants.

Age and BMI- matched healthy controls were recruited by internet advertisements from the local population and underwent the same screening procedures as patients. The inclusion criterion for insomniacs was diagnosis of primary chronic insomnia based on clinical history. Exclusion criteria for insomniacs were any other known sleeping disorders but primary chronic insomnia. Exclusion criteria for healthy controls were any kind of sleeping disorders. Importantly, controls and insomniacs with diabetes mellitus, dyslipidaemia (LDL cholesterol >4.9 mmol/l and/or increased fasting triglycerides >1.7 mmol/l), those taking medication interfering with glucose or lipid metabolism, or presenting with any other significant comorbidity and pregnant and breast feeding women were not eligible to participate.

The diagnosis of “primary chronic insomnia” was made using the Diagnostic and Statistic manual of Mental Disorders IV (DSM-IV) criteria [11]. This means the predominant complaint was difficulty initiating or maintaining sleep, or non-restorative sleep, for at least one month. Further on the sleep disturbance had to cause clinically significant distress. It did not occur exclusively during the course of narcolepsy, breathing-related sleep disorder, circadian rhythm sleep disorder, parasomnia or a mental disorder. Additionally the disturbance was not due to the direct physiological effects of a substance or a general medical condition.

To exclude any organic cause for the sleeping disorder or a metabolic disease, a thorough clinical investigation was performed, and screening blood samples were drawn.

To exclude a psychiatric cause for the sleep disorder the Mini-international Neuropsychiatric Interview (M.I.N.I.) [12] and the Beck Depression Inventory (BDI) [13] were performed.

The M.I.N.I. is a short structured diagnostic interview designed to assess psychiatric diagnoses for clinical trials [12].

The BDI is a 21-question multiple choice self-report inventory for measuring the severity of depression [13]. On a global depression score with a possible range of 0-63, a score of >15 indicates the existence of depression.

Patients and controls eligible to participate in the study protocol based on the results of the screening visit were to undergo a polysomnographic study (PSG) with a portable monitoring system (Siesta ®, Compumedics, Abbotsford, Australia) in order to analyze sleep continuity and architecture.

Participants were monitored for two non-consecutive nights in the course of one week. Electrodes for the electroencephalogram were placed according to the international 10/20-system using C3/ C4 positions occipital and A1/ A2 positions postauricular. The polysomnographic study further included a submental electromyogram for muscle tone, an electrooculogram for eye movement and an electrocardiogram for heart rate. Respiration was monitored using two elastic bands placed around abdomen and thorax registering respiratory excursion. Finger oxymetry provided information about blood oxygenation. Limb movements were measured with two electrodes applied to the Musculi tibialis anteriores.

After the PSG installation patients and controls were discharged to spend the night in their usual environment at home. The next morning patients and controls returned and the polysomnograph was removed.

After the first night all participants were manually scored for restless legs syndrome and sleep apnea using the ProFusion PSG software (Compumedics®, Abbotsford, Australia) according to standardized criteria [14].

Participants having a periodic limb movement index (PLM-index) exceeding 15 and those having a PLM-index with arousal exceeding 5 were excluded from our study, assuming the presence of a restless legs syndrome. Participants having a respiratory deficiency index (RDI) exceeding 5 were also excluded from our study since it indicates sleep apnea.

The second night of the patients was manually analysed regarding quality of sleep using the Profusion PSG software (Compumedics®, Abbotsford, Australia). The following items were evaluated: time of sleep, sleep efficiency ([sleep time · 100/ bed time]), number of arousals, wake up time after initiation of sleep, latency of REM-sleep (time between initiation of sleep and first REM epoch), percentage of the various sleep stages, PLM-index, PLM-index with arousal and RDI.

On the evening before the metabolic studies patients were admitted to the Clinical Research Center (CRC) of the University Hospital Basel. A standard dinner was served containing 30 g protein, 90 g carbohydrate and 30 g fat. Participants were asked to remain fasting until the end of the study. After receiving instructions for 12 h urine collection for catecholamine measurements and salivary samples for cortisol measurements at 11.30 p.m. and 06.00 a.m., patients were discharged.

All participants were readmitted to the outpatient department the next morning. Cortisol levels were determined employing a competitive solid phase time-resolved fluorescence immunoassay with fluorometric end point detection (DELFIA; Wallac, Turku, Finnland). Adrenaline and noradrenaline were measured in 12 h urine using high performance liquid chromatography (CoulArray HPLC system, ESA Biosiences, MA, USA).

Plasma metanephrine concentrations were measured in fasting blood samples using high performance liquid chromatography (CoulArray HPLC system, ESA Biosiences, MA, USA).

Magnetic resonance imaging (MRI) and proton magnetic resonance spectroscopy (^1^H MRS) were performed to gain information about liver fat content, the quantity of adipose tissue and intracellular triglyceride concentrations [15], [16], [17].

For measurements of hepatic lipid accumulation and visceral adipose tissue MRI data were acquired on a 1.5 Tesla whole body system. (Magnetom Avanto, Siemens Healthcare, Erlangen, Germany). A 6 element array coil placed over the right lateral abdomen of the patients and appropriate channels of an 8 element spine coil were used for signal reception.

Anatomical MRI was performed in order to position the 2·2·2 cm^3^ voxel used in the localized spectroscopy experiment. Spectroscopic data were acquired using a double spin echo PRESS sequence without water suppression. RF-pulses with optimised excitation profiles were used in order to achieve good voxel localization and to avoid signal contamination from subcutaneous fat. Four signal averages were collected during one breath hold. Spectroscopic data was analysed using the software package NUTS (Acorn NMR Inc., Livermore, USA). The acquisition of six data sets with spin echo times of 30 ms, 40 ms, 50 ms, 60 ms, 70 ms and 80 ms allowed estimation of fat and water contributions and therefore excluded relaxation phenomena. The lipid signal results from chemically shifted proton resonances corresponding to methyl and methylene groups occupying different positions on lipid molecules with frequencies found in the range −140 Hz to −245 Hz with a major peak at about −220 Hz, corresponding to methylene groups in (CH_2_)n acyl chains. The MRI liver fat percentage was then reported as the spin density of the aliphatic^ 1^H signal divided by the sum of the spin densities of aliphatic and water ^1^H signals. To exclude outliers, the spectroscopic experiment described above was performed for two different voxels in each subject.

The amount of visceral adipose tissue was obtained from multislice T1-weighted MR imaging. Fatty tissue appears bright in T1-weighted MR images and can be easily segmented by thresholding. Subcutaneous fat was separated manually from visceral fat. Visceral and subcutaneous fat volumes were calculated by multiplying the number of voxels containing visceral and subcutaneous fat, respectively by the voxel volume.

To assess intramyocellular lipids (IMCL), spectroscopic MRI data were acquired on a 3.0 Tesla scanner (Magnetom Allegra, Siemens, Erlangen, Germany). Participants were placed in supine position; their right leg was positioned in the center of the radiofrequency headcoil. Anatomical T1-weighted MR imaging was performed in order to define the region of interest. Localized proton spectra were collected using a PRESS sequence. A voxel (10×10×12 mm^3^) was placed in the Musculus tibialis anterior trying to avoid macroscopic visible fat accumulations as well as vessel structures. To optimize the homogeneity of the magnetic field volume selective shimming was accomplished [18]. IMCL concentration was calculated as the ratio between the area under the IMCL peak and the area under the water line.

To assess body composition, body impedance analysis (Bodyimpedance Analyzer Model BIA 101, Akern Srl Florence Italy) was performed. Body composition was calculated using the Bodygram software (Akern Srl Florence Italy).

Indirect calorimetry (Deltatrac II, Datex) was used to examine rates of energy production and substrate oxidation. To assess oxygen consumption, carbon dioxide production and the respiratory quotient (RQ) indirect calorimetry (Deltatrac II, Datex) was performed.

An euglycaemic hyperinsulinemic clamp was performed to assess insulin sensitivity of glucose turnover [19].

An indwelling teflon catheter was placed into an antecubital vein for infusions and a retrograde cannula in a hand vein for blood collection. A 3 hour insulin-glucose clamp began with a primed/continuous insulin infusion (20 mU/m^2^/min) and a variable infusion of 20% dextrose to maintain plasma glucose levels at 5.5±0.5 mmol/l. Arterialized blood was collected during the clamp every 5 min for immediate determination of plasma glucose levels using an automated glucose analyzer (2300 STAT Plus, YSI Bioanalytical Products). The portion of metabolized glucose (M) was calculated as follows: M = GINF – s.

GINF was the glucose concentration in the infusate [mg/(kg×min)] and s the space correction factor. Total M ( µmol/ kg/min) was calculated from the means of the four 10 min periods from 150 to 180 min of the euglycaemic hyperinsulinaemic clamp.

The space correction factor (s) was calculated as follows: s = 3.795×(SA/BW)×(G1–G2) SA was the body surface area (m^2^), and BW the body weight (kg), G1 the initial plasma gucose concentration (mmol/l) and G2 the final plasma glucose concentration (mmol/l).

Plasma insulin levels were measured to ascertain a steady state during the euglycaemic hyperinsulinaemic clamp. Plasma C-peptide concentrations were measured to evaluate if the insulin concentration in the infusate was sufficient to suppress endogenous insulin production.

Insulin and C-peptide levels were determined employing solid-phase, two-site chemiluminescent immunometric assays (IMMULITE 2000 Insulin or C-peptide, respecitvely, from Siemens, UK).

Mann Whitney U tests and correlation analyses were performed using statistical software (JMP Statistical Software, SAS Institute Inc); p<0.05 was defined significant. Initially demographic variables were compared between patients with insomnia and healthy controls. In addition, sleep variables were compared to know if patients and healthy controls differed with respect to sleep. All data are expressed as means±SD.

## Results

### Baseline characteristics (Table 1)

**Table 1:** Baseline characteristics.

	Insomniacs	Controls	p-Value
N	13	12	
Age (years)	51.7±8.0	52.8±9.9	0.36
BMI (kg/m^2^)	22.7±2.6	22.4±1.5	0.68
Waist circumference (cm)	78.3±10.2	77.7±7.1	0.81
Systolic blood pressure (mmHg)	125.0±18.0	116.6±8.5	0.43
Diastolic blood pressure (mmHg)	76.1±6.3	81.6±12.0	0.15
Cholesterol (mmol/l)	5.4±1.0	5.5±1.1	0.82
HDL (mmol/)	2.0±0.4	2.1±0.4	0.56
Triglyceride (mmol/l)	0.9±0.3	0.8±0.2	0.58
ASAT (U/l)	25.2±6.3	24.7±4.9	0.97
ALAT (U/l)	26.9±14.6	18±7.3	0.07
γGT (U/l)	25.0±20.7	16.8±5.9	0.23
CRP (mg/l)	3.0±0.7	2.3±1.1	0.15
Beck Depression Inventory (BDI)	3.1±2.3	2.1±2.1	0.31

Mann Whitney U tests were performed; all data are expressed as means±SD.

Age, BMI, waist circumference, blood pressure, serum cholesterol, HDL-cholesterol, triglycerides, ASAT (aspartate transaminase), ALAT (alanine transaminase), GGT (Gamma-glutamyl transferase) and CRP (C-reactive protein) did not differ significantly between 13 women with chronic insomnia and 12 matched healthy control women. Thus, patients and healthy controls were well matched for demographic, anthropometric and biochemical characteristics.

The Mini- International Neuropsychiatric Interview showed throughout negative answers to all questions in all patients and controls enrolled in the study. Thus, a psychiatric disease was excluded in all participants of the study.

The BDI showed no statistically significant difference regarding depressive symptoms.

### Sleep assessment

All patients suffered from primary chronic insomnia according to DSM IV criteria while controls had a normal sleep-wake-cycle.

In the PSG study the wake time after sleep onset (Table 2) was longer in insomniacs than in controls (*p = 0.05*) whereas no statistically significant differences were found between patients and controls with regard to dimensions of both the remaining sleep continuity and sleep architecture (Table 2).

**Table 2:** Parameters of sleep continuity and sleep architecture (polysomnographic study).

	Insominacs	Controls	p-Value
Number of awakenings	12.3±4.7	11.6 ±4.2	0.97
Wake time after sleep onset (min) *	78.0±57.6	45.9 ±41.2	0.05
Sleep onset latency (min)	17.2±21.0	18.0 ±18.3	0.74
Total sleep time (min)	342.1±84.2	328.6 ±94.5	0.80
Sleep efficiency (%)	76.5±17.6	82.2 ±15.9	0.19
Stage REM latency (min)	101.0 ±64.1	93.9 ±59.9	0.45
Wake time (min)	66.2 ±48.7	44.3 ±41.1	0.16
Stage REM (min)	71.5 ±27.3	56.9 ±31.8	0.30
Stage 1 sleep (min)	19.2 ±10.0	13.9 ±8.9	0.13
Stage 2 sleep (min)	202.5±56.3	222.9±94.8	0.58
Stage 3 sleep (min)	25.4 ±13.4	28.8 ±14.5	0.64
Stage 4 sleep (min)	21.3 ±22.7	11.2 ±16.8	0.12
Stage REM (% sleep time)	5.7 ±2.4	5.2 ±5.5	0.10
Stage 1 (% sleep time)	20.7 ±4.4	17.2 ±7.8	0.35
Stage 2 (% sleep time)	60.2 ±6.7	66.0 ±13.0	0.09
Stage 3 (% sleep time)	7.2 ±2.9	8.1 ±6.8	0.93
Stage 4 (% sleep time)	6.0 ±6.1	3.2 ±4.4	0.11

Mann Whitney U tests were performed; all data are expressed as means±SD; *indicates statistical significance.

### Salivary cortisol, urinary catecholamines, plasma metanephrines

**Figure 2:**
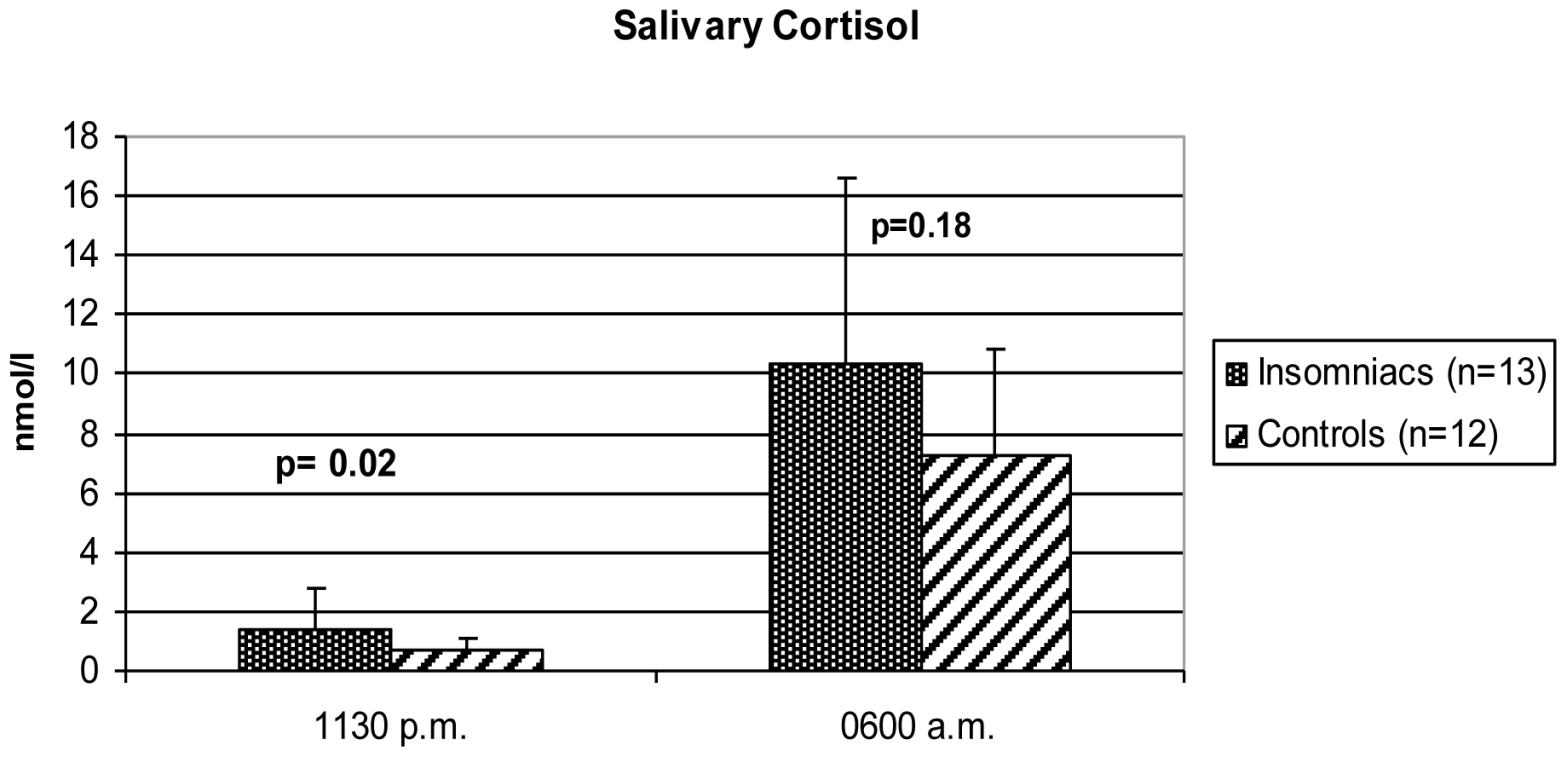
Midnight and morning salivary cortisol concentrations in insomniacs and controls. Midnight values were significantly higher in insomniacs than in controls (p = 0.02), whereas morning values did not differ significantly (p = 0.18).

**Figure 3:**
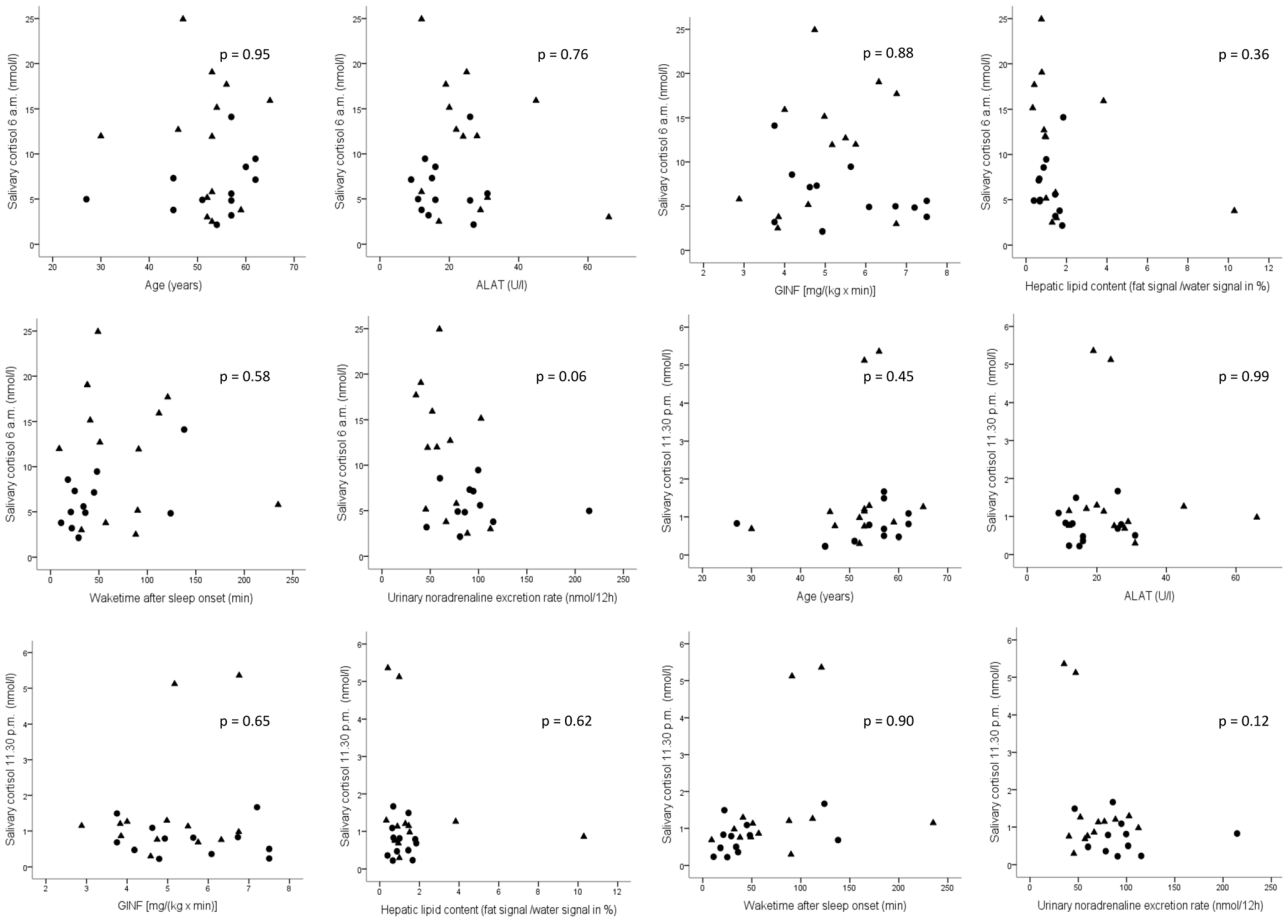
Correlation of salivary cortisol, baseline characteristics, glucose and lipid metabolism as well as catecholamines; filled circles represent controls, filled triangles represent insomniacs; p values including all participants are presented.

Midnight salivary cortisol concentrations were higher in insomniacs compared to healthy controls (*p = 0.02*). No significant differences were observed for morning salivary cortisol concentrations. Urinary 12 h excretion rates of noradrenaline were significantly higher in controls compared to insomniacs (*p = 0.02*), whereas no significant difference was found in urinary excretion rates of adrenalin and dopamine, nor plasma concentrations of metanephrines (Table 3, Fig. 2). No significant correlations between cortisol levels and baseline characteristics, parameters of sleep assessed by PSG, urinary catecholamines, visceral and subcutaneous adipose tissue volume, liver fat content, intramyocellular lipid content and insulin sensitivity in insomniacs alone and in all participants together were identified (Fig. 3).

**Table 3:** Salivary cortisol, urinary catecholamines and dopamine, and plasma metanephrines and 12-hour excretion rates of urinary metanephrines.

	Insomniacs	Controls	p-Value
Salivary cortisol 11:30 p.m. (nmol/l)*	1.4 ±1.4	0.7 ±0.4	0.02
Salivary cortisol 6:00 a.m. (nmol/l)	10.3 ±6.3	7.3 ±3.5	0.18
Urinary adrenaline excretion rate (nmol/12 h)	9.9±11.1	15.5 ±18.1	0.06
Urinary noradrenaline excretion rate (nmol/12 h)*	9.5±23.8	16.0 ±43.4	0.02
Urinary dopamine 12 h excretion rate (nmol/12 h)	13.6±215.2	12.2±294.8	0.64
Plasma normetanephrine (nmol/l)	0.2 ±0.0	0.3 ±0.0	0.15
Plasma metanephrine (nmol/l)	0.1 ±0.0	0.1 ±0.0	0.91

Mann Whitney U tests were performed; all data are expressed as means±SD; *indicates statistical significance.

### MR spectroscopy of liver and muscle

No statistically significant differences of liver fat content, visceral and subcutaneous adipose tissue volume and intramyocellular lipids were found between insomniacs and controls (Table 4).

**Table 4:** Visceral and subcutaneous adipose tissue volume, liver fat content, intramyocellular lipid content (MR spectroscopy) and insulin sensitivity (euglycaemic hyperinsulinaemic clamp).

	Insomniacs	Controls	p-Value
Visceral fat volume (cm^3^)	1140 ±913	793±582	0.47
Subcutaneous fat volume (cm^3^)	4410 ±2558	3476 ±2268	0.32
Hepatic lipid content (fat signal/water signal in%)	1.8 ±2.6	1.0 ±0.5	0.74
Intramyocellular lipid content (IMCL signal/water reference×1000)	4.5 ±2.1	3.9 ±1.51	0.66
M value	4.9±1.1	5.4 ±1.4	0.54
GINF (glucose infusion rate) (mg/kg×min)	5.0 ±1.1	5.5 ±1.4	0.44

Mann Whitney U tests were performed; all data are expressed as means±SD.

### Euglycaemic hyperinsulinaemic clamp technique

No statistically significant difference of insulin-induced glucose uptake, i.e. no difference of insulin sensitivity between patients and controls was found (Table 4).

### Body impedance analysis (BIA) and resting energy expenditure

Healthy controls had a significantly higher amount of total body water when compared to patients (*p = 0.04*). No significant differences in cell mass, weight of fat and non-fat tissue, as well as muscle mass were detected. Respiratory quotients and resting energy expenditure were comparable between patients and controls (Table 5).

**Table 5:** Body composition and resting energy expenditure (measured by BIA and indirect calorimetry, respectively).

	Insomniacs	Controls	p-Value
Total body water (l) *	34.1 ±3.6	37.3 ±2.56	0.04
Cell mass (kg)	26.2 ±3.3	28.6 ±2.4	0.10
Fat tissue (kg)	15.1 ±6.6	11.3 ±6.0	0.11
Non fat tissue (kg)	46.0 ±5.2	50.0 ±3.5	0.10
Muscle mass (kg)	31.6 ±3.8	34.4 ±2.8	0.10
Respiratory quotient	0.9±0.1	0.9±0.1	0.92
Resting energy expenditure (kcal/24 h)	1400.6±180.3	1524.5±132.5	0.10

Mann Whitney U tests were performed; all data are expressed as means±SD; *indicates statistical significance.

## Discussion

The present study investigated whether primary chronic insomnia in otherwise healthy, normal weight women is associated with metabolic and neuroendocrine changes that promote the onset of diabetes. To this aim extensive metabolic phenotyping and sleep studies were performed in 13 women diagnosed with primary chronic insomnia and 12 healthy controls matched for gender, age, BMI, body composition and menopausal status.

Regarding features of the sleep disturbance, it was unexpected to observe that all patients with primary chronic insomnia according to DSM IV criteria showed no major differences in polysomnographic data compared to controls. We only found a significantly higher amount of wake time after sleep onset in patients. There was no significant group difference concerning the various sleep stages. Similar results were found in other studies where only 50–60% of individuals with established diagnostic criteria for insomnia actually showed differences in the polysomnographic studies compared to normal sleepers [20].

A possible explanation for this discrepancy could be the fact that for the traditional PSG scoring a vast amount of data collected during one PSG study is drastically reduced and artificially segmented during analysis. Therefore, the limited representation of sleep monitored by traditional PSG may not fully represent the quality of sleep [20].

Midnight salivary cortisol concentrations were significantly increased in patients with primary chronic insomnia when compared to controls, indicating dysregulation of the hypothalamo-pituitary-adrenal (HPA) axis. Midnight as well as morning salivary cortisol levels were not associated with baseline characteristics, metabolism of glucose and lipids and catecholamines. Similar alterations in evening cortisol levels were observed previously in a study with 11 healthy young men after one night of sleep deprivation [21]. A correlation between elevated evening plasma cortisol levels and sleep deprivation has also been demonstrated in the Whitehall II study, where a cohort of nearly 7000 subjects recruited from the general population was investigated [22]. Further on in infants suffering from infantile colic, fragmented sleep patterns and increased saliva cortisol levels were related [23]. The results of these and our own studies suggest that dysregulation of HPA is a common feature of both acute and chronic sleep curtailment. However, the mechanisms involved are unclear. It is well known that corticotropin-releasing hormone and cortisol have adverse effects on sleep quality [24], [25]. Noteworthy, insomnia can also provoke a stress reaction, including the activation of the HPA-axis [26]. Therefore, it remains unknown whether sleep deprivation leads to increased cortisol levels or if the dysregulation of the HPA-axis causes primary chronic insomnia. Abnormal cortisol levels may precipitate more severe stages of insomnia. However, to proof this hypothesis, longitudinal studies are required.

Interestingly, we found a slightly increased urinary noradrenaline excretion in controls as compared to insomniacs while excretion rates of adrenaline and dopamine and plasma concentrations of metanephrines were not significantly different. These results suggest that the sympatho-adrenal system did not play a pathogenic role in the development of primary chronic insomnia in our patients. Despite extensive investigations employing glucose clamp and in vivo MRI techniques no metabolic features indicating increased diabetes risk or incipient diabetes, such as peripheral insulin resistance or increased intramyocellular or hepatic lipids could be detected in our study population. At a first glance this seems unexpected since several authors reported disturbed glucose metabolism or incident diabetes in patients with short sleep duration [27], [28], sleep disorders such as obstructive sleep apnea [29] and healthy subjects subjected to sleep deprivation [6], [10], [30]. In our study, healthy physically active women with primary chronic insomnia, i.e. subjects reporting difficulty initiating or maintaining sleep, or non-restorative sleep leading to subjective distress and increased wake time after sleep but normal sleep architecture were investigated. The presence of a relatively mild sleep disorder in otherwise healthy women may explain the absence of metabolic disturbances and agrees with the results of Keckeis et al. who could not find impaired glucose tolerance in patients with chronic primary insomnia [31]. Nevertheless, sleep curtailment in subjects having adopted to an unhealthy lifestyle with physical inactivity and ad libitum food intake resulting in weight gain may precipitate impaired glucose metabolism [32].

A limitation of our study may be the relatively small number of participants. However, the variances of the parameters measured were similar to previous studies, and according to our sample size calculation the chance for a statistical error type II was small.

*In summary*, we found that chronic primary insomnia is associated with dysregulation of the HPA-axis, as indicated by increased midnight salivary cortisol concentrations. Although elevated night time cortisol levels have been demonstrated to more profoundly impact glucose metabolism when compared to increased morning cortisol levels [33], we could not demonstrate any changes in glucose and lipid metabolism that may precede the onset of diabetes. Nevertheless, due to the well described detrimental metabolic effects of cortisol oversecretion, the increase in nocturnal cortisol levels may convey an increased risk to develop diabetes and dyslipidema.
